# Trauma Systems in Conflict Zones: A Qualitative Study of Field Operational Requirements in Humanitarian Care

**DOI:** 10.1002/wjs.70322

**Published:** 2026-03-16

**Authors:** Nikolaos Markou‐Pappas, Luca Ansaloni, Luca Ragazzoni, Francesco Barone‐Adesi, Hamdi Lamine

**Affiliations:** ^1^ CRIMEDIM – Center for Research and Training in Disaster Medicine Humanitarian Aid and Global Health Università del Piemonte Orientale Novara Italy; ^2^ Department of Translational Medicine Università del Piemonte Orientale Novara Italy; ^3^ Unit of General Surgery I Fondazione I.R.C.C.S. Policlinico San Matteo Pavia Italy; ^4^ Department of Clinical, Surgical, Diagnostic and Pediatric Sciences University of Pavia Pavia Italy; ^5^ Department for Sustainable Development and Ecological Transition Università del Piemonte Orientale Vercelli Italy; ^6^ Department of Community Health Nursing College of Nursing University of Hail Hail Saudi Arabia

**Keywords:** conflict zones, emergency surgery, humanitarian response, qualitative research, trauma care

## Abstract

**Background:**

Trauma care is a central component of humanitarian medical response in conflict zones. However, essential operational knowledge—referral pathways, triage practices, logistical coordination, and team leadership—remains largely undocumented and inconsistently applied. The absence of structured learning mechanisms perpetuates fragmentation and impedes quality improvement across missions. Our study aimed to capture and analyze the field‐based experiences of humanitarian health professionals to define practical, system‐oriented requirements for effective trauma care in conflict settings.

**Methods:**

We conducted a qualitative, exploratory study grounded in 19 in‐depth, semi‐structured interviews with experienced humanitarian health professionals. Participants were purposively sampled for their experience across prehospital care, hospital‐based trauma response, and humanitarian coordination. Thematic analysis was used to identify structural patterns, operational challenges, and field‐informed strategies that shape trauma care delivery in conflict‐affected contexts. Reporting of this study adhered to the Consolidated Criteria for Reporting Qualitative Research (COREQ).

**Results:**

Participants described trauma care in conflict settings as dependent on interlocking requirements of six interdependent domains. Effective coordination was portrayed not as a technical function but as a relational one, built on trust, preparedness, and shared ownership across agencies and communities. Information exchange needed to be ethically governed, technically reliable, and tailored to fragile environments, relying on simplicity, redundancy, and low‐tech tools co‐developed with local actors. Prehospital care and transport systems were seen as decisive and in need of deliberate design, rooted in safety mapping, role‐adapted responder models, and integration with local infrastructure. Workforce competence extended beyond clinical skills to include cross‐functional agility, cultural literacy, and ethical resilience. Education and training were considered incomplete unless they prepared staff with conflict‐specific competencies, supported by structured, simulation‐based training for both expatriate and local staff. Finally, the absence of embedded operational research was viewed as a critical gap, with respondents calling for real‐time learning systems that inform both frontline response and long‐term planning.

**Conclusion:**

Trauma care in modern conflict cannot rely on improvisation or technical skill alone. It must be underpinned by ethical, resilient and locally grounded systems. Our study highlights the operational knowledge of field practitioners, offering a foundation for building trauma care systems that are integrated, resilient, locally anchored, and worthy of the people they aim to serve.

## Graphical abstract

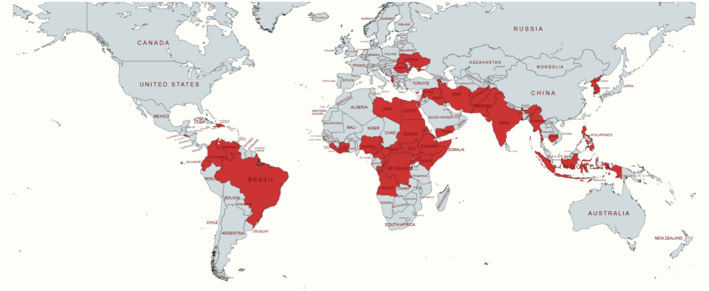

## 1 Introduction

Delivering surgical and emergency care under fire, to civilians and combatants alike, has long stood at the moral and clinical heart of humanitarian response [1, 2, 3]. Yet, despite decades of operations across war‐torn geographies, trauma care remains one of the least standardized, least documented, and most fragmented domains in global health delivery [4, 5].

In stable contexts, trauma systems rely on referral chains, coordinated transport, interoperable communication, and data‐driven oversight [6, 7]. But in conflict zones, these systems fracture. Humanitarian trauma care often unfolds in isolation: disconnected medical posts, improvized triage, and uneven coordination between phases of care [4]. Although chaos is the visible culprit, the deeper issue is the structural marginalization of trauma care as a systems‐level priority within humanitarian health policy and planning [8, 9, 10, 11].

Emerging literature has begun to trace these blind spots, yet much of it remains narrowly focused on clinical or epidemiological dimensions (injury patterns, surgical techniques, or in‐hospital outcomes) [12, 13, 14]. It rarely interrogates the operational architecture that enables care altogether, and most crucially, it omits the voice of those who navigate these breakdowns daily—field practitioners.

In reality, much of humanitarian trauma care knowledge resides in informal, verbal, and experience‐based knowledge, passed from one generation of humanitarians to the next, through handovers between outgoing and incoming expatriate teams, or across hurried briefings in coordination tents [15, 16, 17]. This “grey knowledge,” deeply operational but rarely codified, is the cornerstone of practice in conflict health, a gap in both the academic record and global health policy frameworks.

This study seeks to recenter that frontline intelligence. By drawing on in‐depth interviews with experienced humanitarian healthcare professionals, we aim to synthesize field‐based insights from experienced humanitarian professionals and articulate core operational requirements for trauma system functionality in conflict settings. Although the continuum of trauma care extends to rehabilitation and long‐term recovery, the present study focuses primarily on the acute and systems‐level components of care delivery.

## 2 Methods

We employed a qualitative, exploratory design to surface operational insights from humanitarian trauma care as practiced in conflict zones. Our aim was to draw directly from the lived expertise of health professionals who have navigated, and in many cases, shaped these fragmented care ecosystems.

Data were collected through in‐depth, semi‐structured interviews with 19 humanitarian clinicians and coordinators selected via purposive sampling, intentionally employed to capture information‐rich cases with sustained systems‐level exposure. Recruitment was targeted with direct invitations extended to individuals meeting predefined inclusion criteria designed to reflect the full continuum of trauma system operations. Eligible participants had direct experience at one or more of three operational levels: (1) prehospital care, (2) hospital‐based care (surgical, emergency, critical care), (3) systems‐level coordination, or (4) to involvement in training of humanitarian personnel.

All participants operated within civilian humanitarian mandates, primarily via international NGOs or governmental humanitarian agencies. Individuals with purely military affiliations were excluded to maintain a focus on noncombatant care architectures. A subset was also included based on involvement in workforce training and curriculum development, allowing exploration of institutional knowledge transfer. The final sample reflected a diversity of geographies, roles, and perspectives, ranging from direct field deployment to coordination‐level leadership. Several participants held dual responsibilities in both service delivery and staff preparedness.

Interviews were conducted remotely between October 2023 and March 2024 using Zoom. Each interview was completed in a single sitting and lasted between 60 and 90 min. A semi‐structured guide was designed to elicit both narrative reflections and systems‐level analysis. The tool was piloted with a senior humanitarian surgeon and refined for contextual clarity. The final interview guide is provided as supplementary material. All interviews were conducted in English by a researcher trained in both qualitative methods and humanitarian medicine, audio‐recorded, and transcribed verbatim using Microsoft Word 365.

Thematic analysis followed Braun and Clarke's inductive framework. Coding was conducted iteratively and independently by two researchers using Atlas.ti, allowing themes to emerge from the data rather than being predefined by the interview domains. Discrepancies were resolved through discussion, and themes were refined through constant comparison across transcripts. Reflexive memos were maintained throughout to examine analytic assumptions and decision‐making. Attention was paid to operational patterns, structural constraints, and the intersection between frontline decision‐making and broader organizational logic. Thematic saturation was considered achieved when no substantively new codes or conceptual insights emerged during iterative review.

The research team includes clinicians and scholars with prior experience in humanitarian and conflict health settings. This positionality provided contextual understanding although requiring deliberate reflexivity to mitigate interpretive bias. Trustworthiness was enhanced through dual coding, analytic memoing, audit trails of coding decisions, and regular peer debriefing within the research team.

The study was designed in accordance with humanitarian ethical principles. Ethical approval was secured from the Comitato Etico Territoriale Interaziendale AOU Maggiore della Carità di Novara (ref: CE234/2023). All contributions were anonymized, and informed verbal consent was obtained prior to participation. Data were stored securely in encrypted formats accessible only to the core team. This study was conducted and reported in accordance with the Consolidated Criteria for Reporting Qualitative Research (COREQ) guidelines.

## 3 Results

**FIGURE 1:**
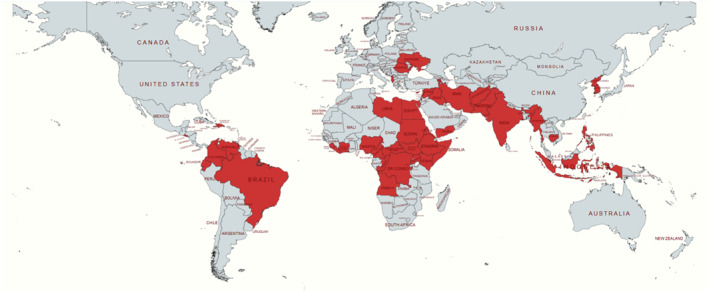
Countries where the participants have been deployed to, facing either active conflict or immediate post‐conflict consequences.

Nineteen humanitarian health professionals participated in this study, bringing experience from sub‐Saharan Africa, the Middle East, and Eastern Europe (Figure 1).

They had a median of 20 years (range: 2–40) of humanitarian field experience and reported between 1 and 12 conflict deployments. Many had operated across multiple levels of trauma systems and across several humanitarian organizations (Table 1).

**TABLE 1:** Participant characteristics.

Professional role	
Trauma/general surgeons	7
Emergency physicians	4
Registered nurses	3
Anesthetists/ICU specialists	2
Paramedics	2
Logistical/Coordination professionals	1
Organizational affiliation	
Médecins Sans Frontières (MSF)	10
International Committee of the Red Cross (ICRC)	
International Federation of the Red Cross (IFRC)	11
EMERGENCY	1
INTERSOS	2
World Health Organization—emergency medical teams (EMTs)	3
Operational domain of experience	
Prehospital care (triage, evacuation, and early stabilization)	6
Hospital‐based surgical (surgical, emergency, and critical care)	16
Systems‐level coordination (referral flows, facility oversight, or operational leadership)	13
Training/curriculum development	10

*Note*: Participants may be counted in more than one category due to overlapping roles, multi‐mission experience, and deployments across multiple regions. Several participants were also involved in pre‐deployment or institutional training within their organizations.

Their testimonies revealed six recurring themes of operational relevance, derived inductively from the data through reflexive thematic analysis. Within each domain, subthemes reflected recurrent operational patterns and shared conceptual emphases across interviews.

### 3.1 Coordination

All participants underscored the role of coordination as central to trauma care delivery in conflict zones. Coordination was described as essential for aligning clinical, logistical, and administrative efforts across all actors involved. Respondents frequently linked coordination gaps with delayed care, resource misallocation, and diminished staff morale.

They emphasized that effective coordination often relied on interpersonal trust rather than institutional structures. Several noted that coordination required humility and recognition of one's limitations. “*Coordination always comes with being humble enough to recognize when your organization is not best placed—and letting someone else do it*,” said one respondent.

Many stressed the importance of trust with local actors, including medical authorities and community leaders. As one emergency physician observed, “*You can't coordinate if you don't have trust*. *And you don't build trust by parachuting with your own protocols and ignoring the local medical staff*.”

Leadership at multiple levels (within clinical teams, across agencies, and at the interface between humanitarian actors and local health authorities) was consistently described as central to coordination effectiveness, particularly if physically present and emotionally attuned. “*You can't lead from behind a laptop*,” one trauma nurse explained. “*You lead by being there when it's hard*, *not just when it's calm*.”

Furthermore, respondents highlighted that trauma systems worked best when built with, not for, local communities. “*Communities don't care where you trained*,” said a field surgeon. “*They care whether you showed up*, *whether you listened*, *whether you respected their space*.”

Examples of co‐designed systems, route mapping, referral planning, and first response, were described as more sustainable and efficient. One humanitarian coordinator emphasized, “*Communities know who needs help*, *who can provide it*, *and how best to do it*. *The requirement is not to teach*; *it's to co‐create*.”

### 3.2 Information Exchange

Participants consistently described information management (clinical, operational, and logistical) between prehospital teams and receiving facilities, across referral centers, among humanitarian agencies, and between field sites and coordinating authorities, as a structural requirement for safe trauma care. Its absence was cited as a recurring vulnerability, contributing to treatment duplication, delays in referral, and poor continuity of care.

Many emphasized the absence of structured handover systems and the resulting fragmentation. “*We treated the same man three times in three different locations*; *each time without knowing his history*,” one emergency surgeon recounted. “*He lost his leg from an infection that could've been managed early*.”

Participants discussed the need for real‐time situational awareness, including availability of beds, surgical capacity, and road safety. The utility of low‐tech, locally managed tools, such as paper triage tags, handwritten referral cards, laminated wristbands, and manual dispatch logbooks, was emphasized repeatedly. Respondents favored consistent systems over sophisticated but fragile technologies. “*The solution isn't fancy software*, *it's consistency*,” one coordinator said.

Several participants drew attention to the disconnect between donor reporting requirements and operational needs. “*We collect data to count beds and admissions*, *but we don't know if the same person walked out or died*,” a respondent stated. The lack of longitudinal data or trauma registries hindered assessment of care outcomes.

Operational data‐sharing platforms were described as unreliable or untrusted. “*We had a shared app*, *but no one updated it because they didn't trust what the others would do with the data*,” noted a medical coordinator. In contrast, systems co‐developed with local staff were more likely to be used and maintained. “*When you give the community ownership*, *they protect the information and use it better*.”

Legal and ethical constraints on data sharing were also discussed. One participant warned, “*A patient list could endanger lives if it fell into the wrong hands*. *But that doesn't mean you abandon documentation*; *it means you need smarter systems*, *not no systems*.”

### 3.3 Prehospital Care and Transport

Participants highlighted prehospital care and transport as domains of high impact and low institutional investment. Care at this level was often improvized, uncoordinated, and shaped by immediate security constraints.

“*The driver is also the medic*. *And the relative*. *And the security*,” noted one physician, referring to the frequent overlap of roles in prehospital scenarios, underscoring the need for field‐ready, role‐expansion training.

Security emerged as a dominant concern. Safe transport required local knowledge, route mapping, and regular coordination with community actors and armed groups. These processes were rarely formalized, and in many cases had to be reestablished during each deployment. “*Transport is a health system function*,” stated a war surgeon. “*It needs to be mapped*, *managed*, *and monitored—not left to chance*.”

Participants called for centralized dispatch systems, adaptable protocols, and transport vehicles suited to both terrain and security risks. Recommendations included the institutionalization of national or regional referral maps and real‐time monitoring of access routes.

### 3.4 Healthcare Workforce Competence

Respondents defined competence as the capacity to act decisively across disciplines and under uncertainty. They emphasized that clinical skill alone was insufficient. “*Cross‐disciplinary practice wasn't a luxury*; *it was the only way we functioned*,” said one nurse manager.

Many described working outside their formal roles. “*One day I was performing laparotomies*; *the next I was fixing our backup generator*,” noted a field surgeon. Flexibility was seen as an operational necessity.

Participants called for structured professional development pathways that gradually exposed clinicians to the realities of fieldwork. Examples included staged deployment models, simulation‐based war surgery courses, and structured mentorship pairings. Credentialism was viewed as inadequate. “*Competence here isn't about the certificate you carry*,” one expert explained. “*It's about the decisions you make when there's no textbook*, *no backup*, *and no second chance*.”

Psychological readiness, cultural humility, and peer support systems were repeatedly highlighted as structural requirements. “*If you think your European medical degree means you know more than the local midwife*, *you will fail*,” said a medical coordinator.

### 3.5 Education and Training

Although some organizations were praised for their war surgery and mass casualty modules, respondents emphasized the absence of a common global curriculum. “*Some people show up brilliant*; *others have never done a fasciotomy or worked without suction*,” one participant said.

Effective training was found to be grounded in realism: simulations with minimal equipment, volatile conditions, and ethical trade‐offs. “*You don't need the best surgeon—you need the best field surgeon*,” a coordinator noted.

Participants called for required cultural orientation, power analysis, and ethics training. Scarcity‐based decision‐making, such as triaging under resource constraint, was cited as emotionally demanding and inadequately covered in most programs. “*We had one bag of IV fluids and five patients*. *Who gets it? Who doesn't? That's not in the manual*,” reflected an emergency physician.

Mentorship was regarded as a key mechanism of learning. “*I learned more from 2 weeks with an old ICRC surgeon than from my whole residency*,” one anesthetist recalled.

### 3.6 Field‐Based Research

Most participants called for research structures that were embedded, continuous, and operationally driven. “*We've been doing this for years*,” said one nurse, “*but when you look for evidence*, *there's nothing but muscle memory*.”

Respondents urged the development of trauma registries capable of tracking patients across care phases. Priorities included evaluating referral chains, infection control, first aid strategies, and rehabilitation.

Several noted the importance of shared ownership, open‐access publication, and research that returns value to the communities it studies. “*We need field‐appropriate answers to field‐born questions*,” summarized one coordinator.

## 4 Discussion

Our study explored field‐based experiences of humanitarian health professionals to identify recurrent operational patterns and systems‐level requirements shaping trauma care delivery in conflict settings. Their insights form a lived blueprint for understanding how trauma systems fracture, and, thus, how they can function when built with intentionality, humility, and interdependence. These findings call for a reframing of humanitarian trauma care not as a clinical challenge alone, but as a systems imperative rooted in preparedness, continuity, and trust.

Coordination emerged as the skeletal architecture of trauma response. Far from a supporting layer, it was described as the mechanism through which care becomes possible. Participants called for coordination to be institutionalized, not activated only in crisis, but embedded in inter‐crisis periods through preparedness planning, codified referral agreements, and real‐time capacity mapping. This aligns with broader global health literature emphasizing the role of pre‐crisis system strengthening and institutionalized governance mechanisms in improving emergency response capacity [18, 19, 20]. Importantly, coordination must be shielded from competition, whether for resources, recognition, or influence, as its sustainability depends on protocols that align incentives across organizations and cultivate shared accountability [21, 22, 23].

Participants emphasized that sustainable coordination requires leadership rooted in proximity, cultural intelligence, and institutional humility. Systems must be designed to function vertically from community to referral centers and horizontally across agencies, ministries, and local authorities. Coordination, to be effective, must move beyond guidelines and become a permanent systems function; one that aligns priorities before the next emergency arrives [18, 20, 24].

Information exchange was described not as a technological issue, but as a structural requirement for safe and equitable trauma care. The challenge lies in designing systems that are trusted, context‐appropriate, and embedded into routine operations, rather than improvized in crisis [25, 26, 27]. Tools must be simple, secure, and redundant, adapted to volatile environments with minimal infrastructure [28]. Respondents cited basic solutions like handwritten tags or radio alerts as more functional than complex platforms, provided they are consistently used and locally owned.

Yet, technology alone is not sufficient. Participants emphasized that trauma information systems must be governed ethically, with safeguards for confidentiality and locally adapted consent processes. Operational transparency must never endanger patients, providers, or communities. To this end, information systems must be part of preparedness, not invented during emergencies, but tested through simulations and supported by inter‐agency agreements [27, 29].

Feedback loops were also seen as essential. Data collection must serve those closest to the patient—providing timely, usable insights that guide real‐time decisions. Information should flow not only upward, but also laterally and back to the field, informing program design and closing the gap between frontline reality and organizational strategy [30]. Trauma care cannot function in silos or in “radio silence”; it must be supported by communication systems that move at the speed of trust [18, 31].

In the domain of prehospital care and transport, participants described a clear principle: definitive trauma care depends on the reliability of upstream systems. It is not surgical teams or ICU beds alone that determine survival, but the transport systems that deliver patients to them [32, 33, 34, 35]. These systems must be deliberately designed, security‐aware, and contextually grounded.

Strategies such as safe passage agreements, marked ambulances, and community‐based triage stations were cited as high‐yield interventions that reduce preventable deaths. Role clarity during mass casualty events (who stabilizes, who documents, and who drives) was emphasized as a minimum requirement for system functionality. Comparable approaches have been documented in prior conflict‐health literature examining access negotiation, protected medical transport, and decentralized triage systems [36, 37, 38, 39, 40, 41]. Importantly, these protocols must be developed with community co‐ownership to ensure legitimacy and adaptability in rapidly shifting environments [42, 43, 44, 45].

Participants challenged the prevailing reliance on in‐hospital metrics as proxies for system success. We propose “survival to arrival” (defined as the proportion of injured patients who reach definitive care alive) as a critical indicator, measuring whether patients reach care in time. Such a shift in metrics would realign priorities toward the full continuum of trauma care and incentivize investment in prehospital systems [46, 47, 48, 49, 50].

Across all interviews, healthcare workforce competence was described as the decisive variable. Yet, they did not define competence by specialty or credentials, but by the agility to perform under uncertainty, adapt protocols, lead diverse teams, and build trust across cultures and disciplines. This broader conceptualization of competency mirrors emerging scholarship in humanitarian workforce development and adaptive leadership in crisis settings [36, 51, 52]. Several participants called for a rethinking of humanitarian workforce models, moving from a rotating pool of volunteers to a standing body of professionals trained in conflict‐specific medicine and systems’ thinking.

We would second such an idea, advocating, in the meantime, for modular training that integrates mentorship, simulation, and progressive field immersion. Such programs should balance technical excellence with leadership, ethics, and cultural humility. To this end, field readiness must be assessed not only by degrees, but also by the capacity to function when there's no textbook, no backup, and no second chance [53, 54, 55, 56].

A recurring theme was the need to bridge the growing divide between “field” and “headquarters” mindsets. Several advocated for dual‐track deployments, combining frontline clinical rotations with coordination roles, to foster mutual understanding and policy grounded in operational truth. This, they argued, would enhance not only effectiveness, but also professional resilience and retention.

Crucially, participants emphasized that any model of preparedness or professionalization must elevate local clinicians and systems as the primary custodians of trauma care. Training models must therefore be inclusive, sustainable, and designed for shared ownership [42, 57, 58].

Finally, across all domains, participants expressed a shared concern: trauma care in conflict zones remains too reliant on experiential memory and too weak in structured evidence. Without embedded research and longitudinal data, programs measure what is visible—bed occupancy and procedures performed—but not what matters the most: recovery, dignity, reintegration, and long‐term functionality [59, 60].

Participants called for operational research that is context‐driven, locally anchored, and inseparable from care delivery. Research must respond to practical questions—how to triage in insecurity, how to manage antibiotics without labs, and how to delegate safely across cadres—and must be structured to generate usable insights in real time [59, 61, 62].

Embedded research also demands an ethos of humility. Communities must be engaged not only as data points, but as partners and co‐designers. Findings must be shared back, translated into action, and grounded in ethical practice [42, 61]. In fragile environments where trauma systems collapse when international teams withdraw, research must focus on governance, financing, and models of care that can endure instability [58, 60, 62].

Sustainable, ethical trauma care in war zones requires more than clinical skill—It requires systems that are locally owned, evidence‐informed, and designed to function across the full arc of the patient journey. That is the imperative our participants outlined. And that is the challenge this study seeks to advance.

### 4.1 Limitations

This study is limited by the sample size, which, although diverse in geography and experience, cannot claim representativeness of the broader humanitarian workforce. Participants were selected through purposive sampling and based on availability and self‐reported roles, which may introduce selection bias. Operational constraints also meant interviews were conducted remotely, limiting contextual observations. In addition, although trauma systems extend into rehabilitation and long‐term follow‐up, post‐discharge and rehabilitative care were beyond the scope of this analysis. Furthermore, although the study prioritized depth and authenticity of field perspectives, it does not include voices of national responders or affected community members, a gap that warrants attention in future research. Finally, the research team's prior experience in humanitarian health, while providing contextual insight, may have influenced analytic framing and interpretation despite deliberate reflexive practices.

## 5 Conclusion

These insights are not universal prescriptions. They are an appeal for operational realism, ethical clarity, and a shared ambition to build the foundational architecture of trauma care systems in modern conflict settings.

Our findings urge humanitarian actors, NGOs, ministries of health, donors, and academia, to move beyond episodic interventions toward locally led and globally supported, integrated, resilient trauma systems. Such shift demands that we redesign how trauma care is conceived, staffed, and measured—elevating systems thinking alongside clinical expertise [63, 64, 65, 66].

The operational challenges described by field practitioners are not barriers to be endured; they are the scaffolding upon which more just, responsive systems can be built. We must go beyond life and limb emergency frontline mentality toward a comprehensive integrated system‐centered approach.

## Author Contributions

**Nikolaos Markou‐Pappas:** conceptualization, data curation, formal analysis, investigation, methodology, software, validation, visualization, writing – original draft, writing – review and editing. **Luca Ansaloni:** formal analysis, methodology, validation, visualization, writing – original draft, writing – review and editing. **Luca Ragazzoni:** conceptualization, supervision. **Francesco Barone‐Adesi:** conceptualization, supervision, writing – review and editing. **Hamdi Lamine:** conceptualization, formal analysis, investigation, methodology, software, supervision, validation, visualization, writing – original draft, writing – review and editing.

## Funding

The authors have nothing to report.

## Conflicts of Interest

The authors declare no conflicts of interest.

## Supporting information


Supporting Information S1

## Data Availability

The data that support the findings of this study are available from the corresponding author upon reasonable request.
