# Supplementary material for: Trauma Systems in Conflict Zones: A Qualitative Study of Field Operational Requirements in Humanitarian Care
Source: World J Surg. 2026 Mar 16;50(4):927–35. doi: 10.1002/wjs.70322 (PMC13070445; doi:10.1002/wjs.70322)
Supplement: Supplementary file 1 — Supporting Information S1 [file WJS-50-927-s001.docx]

**Trauma Systems in Conflict Zones: A Qualitative Study of Field Operational Requirements in Humanitarian Care**

Considering these growing trends and the ongoing need to "push" humanitarian actors ever closer to the frontline, revising the requirements and procedures for providing frontline trauma care to the affected people becomes necessary. This research project aims to advance the general knowledge of civilian trauma system implementation in conflict settings by mapping out challenges in past conflicts and past solutions put in place to address them. Furthermore, bringing together specialists with a range of perspectives to settle on a widely acknowledged framework to aid in identifying such gaps will inform and help strengthen future responses in a pragmatic fashion.

1. Can you provide some information about yourself: Professional background, Years of experience in the humanitarian field, Number of participation in humanitarian missions in conflict settings, which conflict settings?
2. In your opinion, what are the elements of effective coordination among different stakeholders involved in trauma care provision?

Prompt: What recommendations do you have for improving coordination among stakeholders within a trauma system?

1. What communication strategies are important for ensuring timely and accurate information exchange within a trauma system?

Prompt: What communication methods do you find most effective in these environments?

1. From your perspective, what are the components of an efficient transportation system within a trauma system?

Prompt: According to you, how can timely access care for trauma patients through appropriate transportation to healthcare facilities can be guaranteed?

1. What are the features that an ideal patient health information system for trauma care should have?

Prompt: According to you, how can its adaptation among healthcare facilities and providers enhance the continuity of care for trauma patients?

1. What specific competencies are important for healthcare professionals working within a trauma system to have?
2. What recommendations do you have for designing and implementing ongoing education and training programs for trauma care providers?
3. What do you see as the most pressing research priorities in the field of trauma care provision by humanitarian actors in conflict settings?
4. Do you have anything else to add?
